# Sustained poor mental health among healthcare workers in COVID‐19 pandemic: A longitudinal analysis of the four‐wave panel survey over 8 months in Japan

**DOI:** 10.1002/1348-9585.12227

**Published:** 2021-05-22

**Authors:** Natsu Sasaki, Hiroki Asaoka, Reiko Kuroda, Kanami Tsuno, Kotaro Imamura, Norito Kawakami

**Affiliations:** ^1^ Department of Mental Health Graduate School of Medicine The University of Tokyo Tokyo Japan; ^2^ Department of Psychiatric Nursing Graduate School of Medicine The University of Tokyo Tokyo Japan; ^3^ Division for Environment, Health, and Safety The University of Tokyo Tokyo Japan; ^4^ School of Health Innovation Kanagawa University of Human Services Kanagawa Japan

**Keywords:** anxiety, depression, public health, stress

## Abstract

**Objectives:**

This study aimed to compare the longitudinal change of the psychological distress of healthcare workers (HCWs) with non‐HCWs during the repeated outbreaks of the COVID‐19 in Japan.

**Methods:**

The data were retrieved from the Employee Cohort Study in the Covid‐19 pandemic in Japan study. An online survey was conducted on March 2020 (T1), on May 2020 (T2), on August 2020 (T3), and on November 2020 (T4). Psychological distress was measured by the Brief Job Stress Questionnaire. A mixed‐model repeated‐measures ANOVA was conducted as an indicator of the group differences.

**Results:**

A total sample of analysis was n = 996 (HCWs, n = 111; non‐HCWs, n = 885). HCWs consisted of physicians/nurses/midwives and other HCWs (eg, pharmacists, clinical laboratory technicians) in the clinical settings (n = 19; 17% and n = 61; 55%, respectively), and HCWs not working in the clinical settings (n = 31; 28%). Being HCWs were associated with a significant increase in psychological distress from T1 to T2, T3 and T4 (*P* = .001, *P* = .002, *P* < .001; respectively).

**Conclusions:**

The mental health of HCWs deteriorated through the COVID‐19 outbreaks compared with non‐HCWs. HCWs are continuously the important targets to provide mental health support.

## 1 INTRODUCTION

Healthcare workers (HCWs) are at high risk of worsened mental health under the COVID‐19 pandemic. Systematic reviews have revealed that HCWs experienced poor mental health such as depression, anxiety, burnout, insomnia, post‐traumatic stress reactions, and distress.^1^, ^2^ Poor mental health also has been reported among not only frontline doctors and nurses but also second‐line HCWs (without direct contact of COVID‐19) and other co‐medicals.^3^, ^4^, ^5^ In cross‐sectional studies, mental health has been reported to be even poorer among HCWs than among other occupations at one point of time during the outbreak.^6^, ^7^ Our previous longitudinal study found that psychological distress deteriorated more among HCWs compared with non‐HCWs among full‐time employees during the first wave of the COVID‐19 outbreak in Japan (March‐May 2020).^8^ However, it is unknown whether level of psychological distress among HCWs changed over repeated outbreaks of COVID‐19. HCWs may have had improved mental health in between the outbreaks or they may have sustained poor mental health despite the changing levels of the pandemic because they were on alert preparing for outbreaks. Such a finding would be useful for understanding the long‐term psychological burden related to COVID‐19 among HCWs. It may be also useful in planning mental health countermeasures for HCWs during COVID‐19 outbreaks. Using a four‐wave longitudinal panel data collected from an early phase of the first outbreak (March 2020) until an early phase of third outbreak (November 2020) of COVID‐19 in Japan, this study investigated how the mental health of the HCWs deteriorated over time during repeated COVID‐19 outbreaks, compared with non‐HCWs.

## 2 METHODS

### 2.1 Study design and participants

The data were retrieved from the Employee Cohort Study in the Covid‐19 pandemic in Japan (E‐COCO‐J).^8^, ^9^ Full‐time employees recruited from the panel of the Japanese internet company completed an online baseline survey during March 19‐22, 2020 (n = 1448). Respondents, excluding unemployed (n = 27), were invited to complete the survey on May 22‐26, 2020 (T2), and on August 7‐12, 2020 (T3). The respondents who answered the baseline (n = 1448) were invited to complete the survey on November 6‐12, 2020 (T4).

The details of the recruitment process are shown in Figure S1. Participants who were currently working or temporarily laid off were included as an analytic sample. The time frame between the survey and COVID‐19 situations (ie, polymerase chain reaction positive cases and severe patients) is shown in Figure S2. This study was approved by the Research Ethics Committee of the Graduate School of Medicine/Faculty of Medicine, The University of Tokyo (no. 10856‐(2)(3)(4)(5)).

### 2.2 Measurement variables

#### 2.2.1 Psychological distress

Psychological distress in the last 30 days was measured by the 18‐items scale included in the 57‐item version of Brief Job Stress Questionnaire,^10^ a multidimensional questionnaire that measures various types of job stressors, psychological and physical symptoms, and workplace support. All items were rated by using a four‐point Likert scale from 1 (*almost never*), 2 (*sometimes*), 3 (*often*), and 4 (*almost always*). The scores were summed, with higher scores indicating greater distress. The possible score range was from 18 to 72. Cronbach's alpha coefficients of the internal consistency reliability of the psychological distress scale in this sample were: 0.934 at T1, 0.928 at T2, 0.938 at T3, and 0.936 at T4.

#### 2.2.2 HCWs or non‐HCWs

Respondents were asked about their current occupations and the facilities at T2. The response options were as follows: I am (i) non‐HCW, (ii) physician, (iii) nurse/midwife, (iv) other HCW (eg, pharmacist, clinical laboratory technician) working in healthcare facilities, and (v) HCW but not working in healthcare settings. Participants were divided into two categories: non‐HCWs (i) and all types of HCW (ii)‐(v). Besides, the HCWs were dichotomized into two categories: clinical (ii)‐(iv) and non‐clinical (v).

#### 2.2.3 Demographic variables

We measured sex, age, marital status, and educational attainment (≥16 years) as covariates in statistical analysis. We also collected information on industry and organization size. All the demographic variables were retrieved at T1 except education (T2).

### 2.3 Statistical analysis

A mixed‐model repeated‐measures ANOVA with an unstructured covariance matrix was conducted using a group (HCW vs non‐HCW) × time (T1, T2, T3, T4) interaction as an indicator of the group differences. The outcome was treated as a missing variable if participants were unemployed, on sick leave, on temporary leave, or on maternity leave at every time point in the mixed‐model analysis. This model handled and imputed missing data with restricted maximum likelihood estimation assuming missing at random. As a sensitivity analysis, we conducted the same analysis among HCW between clinical and non‐clinical groups. SPSS 26.0 (IBM Corp) Japanese version was used. Statistical significance was set as a two‐sided *P* < .05. Adjustment for multiple comparisons was not made, because all the statistical tests were conducted simultaneously in a regression model.

## 3 RESULTS

The total number of analytic samples was 996 after excluding unemployed (n = 17), sick leave (n = 2), and maternity leave (n = 17) at T2. HCWs (n = 111) and non‐HCWs (n = 885) were included. Participants' characteristics are listed in Table S1. HCWs were more female and less educated. HCWs consisted of physicians/nurses/midwives and other HCWs (eg, pharmacists, clinical laboratory technicians) in the clinical settings (n = 19; 17% and n = 61; 55%, respectively), and HCWs not working in the clinical settings (n = 31; 28%).

The mean scores of psychological distress at each time point are given in Table 1. The results of a mixed model ANOVA showed that being a HCW was associated with a significant increase in psychological distress from T1 to T2, T3, and T4 compared with non‐HCWs (adjusted estimates of fixed effect 2.89 [95% CI 1.23‐4.55], *P* = .001; 2.87 [1.09‐4.65], *P* = .002; 3.76 [1.90‐5.63], *P* <.001). Details are given in Table 2. The main effect of HCWs compared with non‐HCWs was not significant for psychological distress (adjusted estimates of fixed effect −1.73 [−4.01 to 0.55], *P* = .137). The overall main effect of time was significant (*df* = 3, *P* < .001): compared from T1, adjusted estimates of fixed effect were −0.27 [−0.83 to 0.28], *P* = .334, for T2; 0.61 [0.028 to 1.19], *P* = .040, for T3; and −0.50 [−1.09 to 0.085], *P* = .093, for T4.

**TABLE 1:** The crude means of psychological distress at baseline (T1), T2, T3, and T4 under COVID‐19 pandemic among the cohort of Japanese employees stratified into healthcare workers (HCWs) and non‐HCWs (N = 996)

Survey (time of survey)	Total N^b^	HCWs^a^			Non‐HCWs^a^		
		n	Mean	SD	n	Mean	SD
T1 (March 2020)	996	111	40.2	10.9	885	41.4	11.7
T2 (May 2020)	968	108	42.9	11.8	860	41.0	11.0
T3 (August 2020)	894	95	43.6	12.3	799	42.0	11.7
T4 (November 2020)	864	83	43.9	11.9	781	40.9	11.4

Abbreviations: COVID‐19, coronavirus disease 2019; SD, standard deviation.

^a^ The information about HCWs or non‐HCWs was measured on T2. Healthcare workers included physicians, nurses, midwives, other healthcare workers (eg, pharmacists, clinical laboratory technicians), and HCWs but not working in clinical settings.

^b^ The outcome was treated as a missing variable if participants were unemployed, on sick leave, on temporary leave, or on maternity leave at every time point.

**TABLE 2:** The crude and adjusted estimated mean of psychological distress at baseline (T1), T2, T3, and T4 under COVID‐19 pandemic among the cohort of Japanese full‐time employees: the mixed model with repeated measures (N = 996)

Survey (time of survey)	Crude			Adjusted^a^		
	HCWs^b^	Non‐HCWs^b^	Survey × group interaction	HCWs^b^	Non‐HCWs^b^	Survey × group interaction
	Estimated mean (SE)	Estimated mean (SE)	*P* value	Estimated mean (SE)	Estimated mean (SE)	*P* value
T1 (March 2020)	40.2 (1.1)	41.4 (0.4)	Ref	39.8 (1.1)	41.6 (0.4)	Ref
T2 (May 2020)	42.8 (1.1)	41.1 (0.4)	0.001* (T1 vs T2)	42.5 (1.1)	41.3 (0.4)	0.001* (T1 vs T2)
T3 (August 2020)	43.7 (1.1)	42.0 (0.4)	0.002* (T1 vs T3)	43.3 (1.1)	42.2 (0.4)	0.002* (T1 vs T3)
T4 (November 2020)	43.4 (1.2)	40.9 (0.4)	<0.001** (T1 vs T4)	43.1 (1.2)	41.1 (0.4)	<0.001** (T1 vs T4)

Abbreviations: COVID‐19, coronavirus disease 2019; SE, standard error.

^a^ Adjusted for age (20‐29, 30‐39, 40‐49, or over 50 y old), gender, marital status (single or married), and education attainment (≥16 y or less).

^b^ The information about HCWs or non‐HCWs was measured on T2. Healthcare workers included physicians, nurses, midwives, other healthcare workers (eg, pharmacists, clinical laboratory technicians), and HCWs but not working in clinical settings.

* *P* < .05,

** *P* < .001.

As a sensitivity analysis, the clinical group of HCWs was not associated with increased psychological distress from T1 to T2, T3, and T4 compared with non‐clinical HCWs after adjusting all covariates (data available upon request).

## 4 DISCUSSION

The result of this longitudinal analysis showed that after psychological distress in HCWs increased during the first wave of the COVID‐19 outbreak in Japan from March to May 2020,^8^ it remained elevated at a same level in August and November 2020, while the epidemic of COVID‐19 had breaks between the repeated outbreaks. The pattern was in contrast to that among non‐HCWs: psychological distress was significantly greater among HCWs than non‐HCWs at the T2‐T4 surveys between May and November 2020. The T4 survey was conducted in the early phase of the third wave of pandemic, leading to an underestimation of psychological distress among HCWs. The results suggested that the outbreaks (ie, increased number of patients) had a greater psychological impact on HCWs than on non‐HCWs. The results also indicated that HCWs continued to be at risk and in need of mental health prevention and promotion at the workplace even between outbreaks of COVID‐19. A closer monitoring of and active intervention for the mental health of HCWs should be continued.

The study demonstrated that elevated psychological distress among HCWs was present not just in the initial phase (the first wave) of the COVID‐19 outbreak but also was sustained at a similar level during and between repeated outbreaks.^11^ The finding agrees with previous cross‐sectional studies of poor mental health of HCWs at one point in time during the COVID‐19 outbreak,^6^, ^7^ but adds evidence that HCWs continuously suffered from high psychological distress during repeated outbreaks. People with non‐clinical psychological distress or depression have longer sickness days, poor functioning at home and work, and greater risk of having an overt mental disorder (such as major depression) and health care utilization^12^; moreover a longer duration of distress is associated with poorer outcomes.^13^ Psychological distress sustained over 6 months could have a non‐negligible impact on quality of life of HCWs. Possible reasons for elevated psychological distress continuing despite the fluctuation of outbreaks might be that HCWs—both front‐line and non‐front line HCWs—had to remain on guard against unexpected contacts, and to face the risk of infection and transmission to family members^4^, ^14^ In addition, HCWs suffered conditions including stigmatization and discrimination, lack of social support from family and friends, lack of psychological reward under high demands at work, and difficulty in using effective coping strategies. As with the psychological symptoms of distress, such conditions continued under the repeated outbreaks.

The findings indicated that medical institutions/facilities should remain alert to the potential for HCWs to experience deteriorated mental health as COVID‐19 outbreaks wax and wane, while implementing consensus‐based measures to support the mental health of HCWs.^15^ Moreover, because ongoing psychological distress may be associated with a greater risk of sickness absence, burnout, and mental disorders,^12^, ^13^ implementation of more active countermeasures also should be considered. The findings suggested that HCWs in non‐clinical settings can be in need of focused mental health care as well.

This study has several limitations. The use of an online survey might cause selection bias. The relatively small sample of HCWs, especially the small number of physicians and nurses in clinical settings, cannot be regarded as a representative HCW sample. The generalization of the present findings should be careful. Besides, it may lead to an underestimation of the psychological distress in HCWs. The analysis did not consider the number of positive COVID‐19 cases at each point and area distribution; however, the pandemic had prevailed in throughout Japan at T4 (November 2020). HCWs may have more difficulties in daily life in a COVID‐19 outbreak, such as taking care of their children when nurseries and schools were closed, or commuting to work with crowded public transportation because of a limited chance to work from home, than non‐HCWs, which may explain the observed difference in psychological distress between the two groups. We could not explore these possibilities because we did not measure these experiences.

## 5 CONCLUSION

Healthcare workers continuously experienced high psychological distress in August and November 2020 in this four‐wave longitudinal panel study of employees in Japan (E‐COCO‐J), despite changing levels of the epidemic of COVID‐19, after it increased during the first‐wave outbreak in May 2020.^8^ The levels of psychological distress were consistently significantly higher among HCWs than among non‐HCWs at these surveys. A closer monitoring of and active intervention for the mental health of HCWs should be continued.

## DISCLOSURE

*Approval of the research protocol*: This study was approved by the Research Ethics Committee of the Graduate School of Medicine/Faculty of Medicine, The University of Tokyo, No. 10856‐(2)(3)(4)(5). *Informed consent*: Online informed consent was obtained from all participants with full disclosure and explanation of the purpose and procedures of this study. We explained that their participation was voluntary, and they can withdraw consent for any reason, simply by not completing the questionnaire. *Registry and registration number of the study/trial*: N/A. *Animal studies*: N/A. *Conflict of interest*: NK reports grants from SB AtWork Corp, Fujitsu Ltd, and TAK Ltd, personal fees from the Occupational Health Foundation, SB AtWork Corp, RIKEN, Japan Aerospace Exploration Agency (JAXA), Japan Dental Association, Sekisui Chemicals, Junpukai Health Care Center, Osaka Chamber of Commerce and Industry, outside the submitted work.

## AUTHOR CONTRIBUTION

NK was in charge of this study, supervising the process, and of providing his expert opinion. NS, HA, and NK organized the study design and analyzed the data. Collaborators RK, KT, and KI ensured that questions related to the accuracy or integrity of any part of the work were appropriately investigated and resolved. All authors participated in conducting the survey. NS and NK wrote the first draft of the manuscript, and all other authors critically revised it. All authors approved the final version of the manuscript.

## ROLE OF THE FUNDER/SPONSOR

The sponsors had no role in the design and conduct of the study; collection, management, analysis, and interpretation of the data; in the preparation, review, or approval of the manuscript; and in the decision to submit the manuscript for publication.

## Supporting information

Supplementary Materials

Supplementary Materials

Supplementary Materials

Fig S1

Fig S2

Table S1
