# Supplementary material for: Sustained poor mental health among healthcare workers in COVID‐19 pandemic: A longitudinal analysis of the four‐wave panel survey over 8 months in Japan
Source: J Occup Health. 2021 May 22;63(1):e12227. doi: 10.1002/1348-9585.12227 (PMC8140377; doi:10.1002/1348-9585.12227)
Supplement: Supplementary file 1 — Supplementary Materials [file JOH2-63-e12227-s006.pdf]

**Supplementary Table S1. Baseline (T1) characteristics of participants (N=996).**

| | Healthcare Workers (HCWs) [a] (n=111) | | Non- HCWs[a] (n=885) | | P for difference [b] |
|---|---|---|---|---|---|
| | N (%) | Mean (SD) | N (%) | Mean (SD) | |
| Gender | | | | | <0.001 |
| Male | 39 (35.1) | | 469 (53.0) | | |
| Female | 72 (64.9) | | 416 (47.0) | | |
| Age | | 39.6 (10.6) | | 41.5 (10.5) | 0.078 |
| 20-29 years old | 24 (21.6) | | 162 (18.3) | | 0.323 |
| 30-39 years old | 36 (32.4) | | 234 (26.4) | | |
| 40-49 years old | 24 (21.6) | | 236 (26.7) | | |
| Over 50 years old | 27 (24.3) | | 253 (28.6) | | |
| Marital status | | | | | 0.797 |
| Single | 57 (51.4) | | 443 (50.1) | | |
| Married | 54 (48.6) | | 442 (49.9) | | |
| Education attainment [c] | | | | | <0.001 |
| Junior high school | 0 | | 7 (0.8) | | |
| High school | 16 (14.4) | | 216 (24.4) | | |
| Vocational/College | 56 (50.5) | | 182 (20.6) | | |
| University | 36 (32.4) | | 429 (48.5) | | |
| Graduate university | 3 (2.7) | | 51 (5.8) | | |
| Company size | | | | | 0.004 |
| >1000 employees | 22 (19.8) | | 301 (34.0) | | |
| 300-999 | 28 (25.2) | | 146 (16.5) | | |
| 50-299 | 30 (27.0) | | 237 (26.8) | | |
| <50 | 31 (27.9) | | 173 (19.5) | | |
| unknown | 0 | | 28 (3.2) | | |
| Healthcare worker details | | | | | |
| Physicians | 4 (3.6) | | | | |
| Nurses/midwives | 15 (13.5) | | | | |
| Other healthcare workers (e.g., pharmacists, clinical laboratory technicians) | 61 (55.0) | | | | |

| | |
|---|---|
| Health care workers but not working in clinical settings | 31 (27.9) |

SD: standard deviation.

[a] The information about health care workers or general workers was measured on T2. Health care workers included physicians, nurses, midwives, other health care workers (e.g., pharmacists, clinical laboratory technicians), and health care workers but not working in clinical settings.

[b] P value for difference was calculated by chi square test for variables except for age. T test was used for age.

[C] The education attainment was measured at T2.

**Table 1. The crude means of psychological distress at baseline (T1), T2, T3, and T4 under COVID-19 pandemic among the cohort of Japanese employees stratified into healthcare workers (HCWs) and non-HCWs (N=996).**

| | | HCWs [a] | | | Non-HCWs [a] | | |
|---|---|---|---|---|---|---|---|
| Survey (time of survey) | Total N [b] | n | Mean | SD | n | Mean | SD |
| T1 (March 2020) | 996 | 111 | 40.2 | 10.9 | 885 | 41.4 | 11.7 |
| T2 (May 2020) | 968 | 108 | 42.9 | 11.8 | 860 | 41.0 | 11.0 |
| T3 (August 2020) | 894 | 95 | 43.6 | 12.3 | 799 | 42.0 | 11.7 |
| T4 (November 2020) | 864 | 83 | 43.9 | 11.9 | 781 | 40.9 | 11.4 |

COVID-19: Coronavirus disease 2019.

SD: standard deviation.

[a] The information about HCWs or non-HCWs was measured on T2. Health care workers included physicians, nurses, midwives, other health care workers (e.g., pharmacists, clinical laboratory technicians), and HCWs but not working in clinical settings.

[b] The outcome was treated as a missing variable if participants were unemployed, on sick leave, on temporary leave, or on maternity leave at every time point.

**Table 2. The crude and adjusted estimated mean of psychological distress at baseline (T1), T2, T3, and T4 under COVID-19 pandemic among the cohort of Japanese full-time employees: the mixed model with repeated measures (N=996).**

| | Crude | | | Adjusted [a] | | |
|---|---|---|---|---|---|---|
| Survey (time of survey) | HCWs [b] | Non-HCWs [b] | Survey x group interaction | HCWs [b] | Non-HCWs [b] | Survey x group interaction |
| | Estimated mean (SE) | Estimated mean (SE) | p value | Estimated mean (SE) | Estimated mean (SE) | p value |
| T1 (March 2020) | 40.2 (1.1) | 41.4 (0.4) | ref | 39.8 (1.1) | 41.6 (0.4) | ref |
| T2 (May 2020) | 42.8 (1.1) | 41.1 (0.4) | 0.001* (T1 vs T2) | 42.5 (1.1) | 41.3 (0.4) | 0.001* (T1 vs T2) |
| T3 (August 2020) | 43.7 (1.1) | 42.0 (0.4) | 0.002* (T1 vs T3) | 43.3 (1.1) | 42.2 (0.4) | 0.002* (T1 vs T3) |
| T4 (November 2020) | 43.4 (1.2) | 40.9 (0.4) | <0.001** (T1 vs T4) | 43.1 (1.2) | 41.1 (0.4) | <0.001** (T1 vs T4) |

COVID-19: Coronavirus disease 2019. * $p<0.05$, ** $p<0.001$.

SE: standard error.

[a] Adjusted for age (20-29, 30-39, 40-49, or over 50 years old), gender, marital status (single or married), and education attainment (≥16 years or less).

[b] The information about HCWs or non-HCWs was measured on T2. Health care workers included physicians, nurses, midwives, other health care workers (e.g., pharmacists, clinical laboratory technicians), and HCWs but not working in clinical settings.
