# Supplementary figures and images for: Sustained poor mental health among healthcare workers in COVID‐19 pandemic: A longitudinal analysis of the four‐wave panel survey over 8 months in Japan
Source: J Occup Health. 2021 May 22;63(1):e12227. doi: 10.1002/1348-9585.12227 (PMC8140377; doi:10.1002/1348-9585.12227)

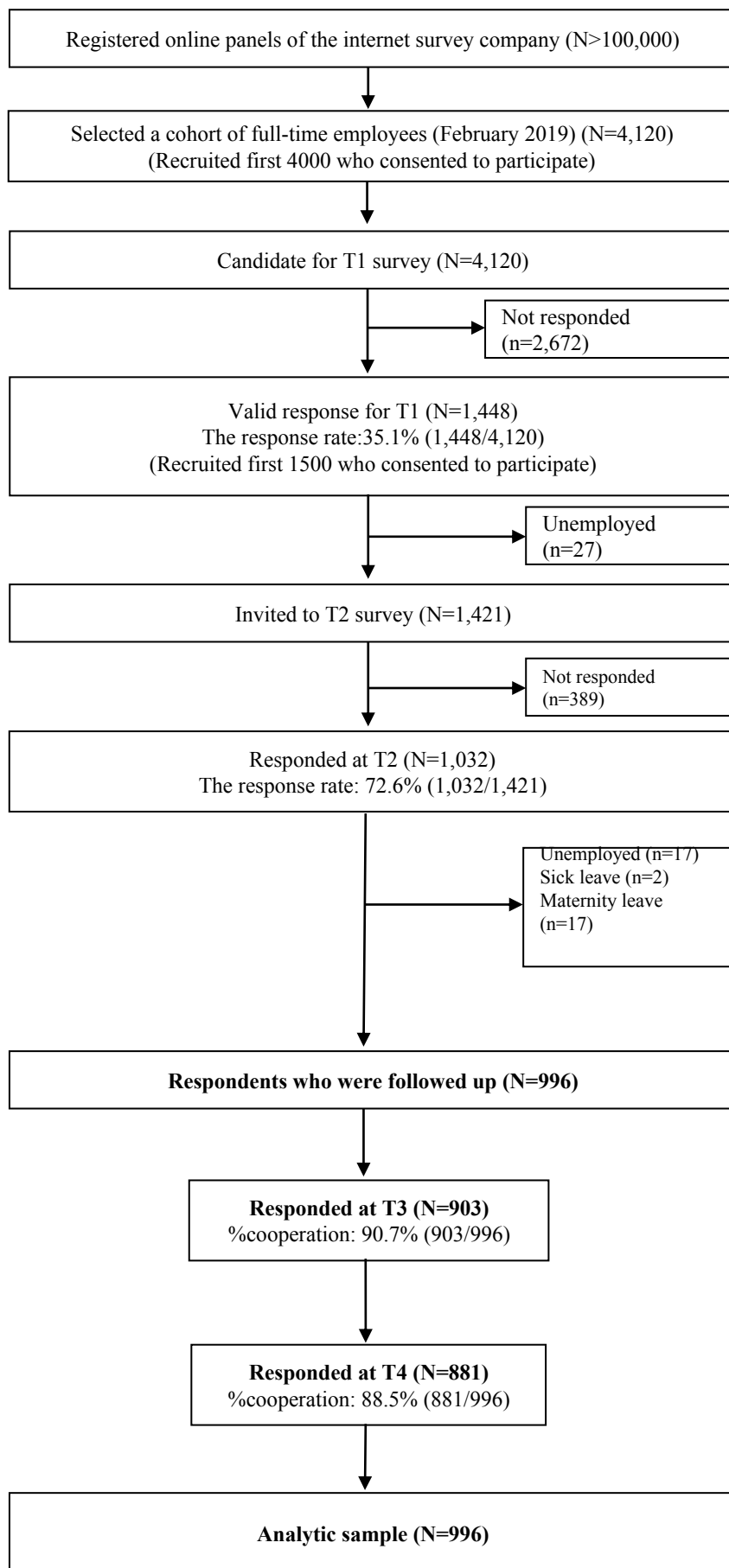

Supplementary figure 1. Flowchart of participant recruitment.

Supplement: Supplementary file 2 — Supplementary Materials [file JOH2-63-e12227-s002.pdf]

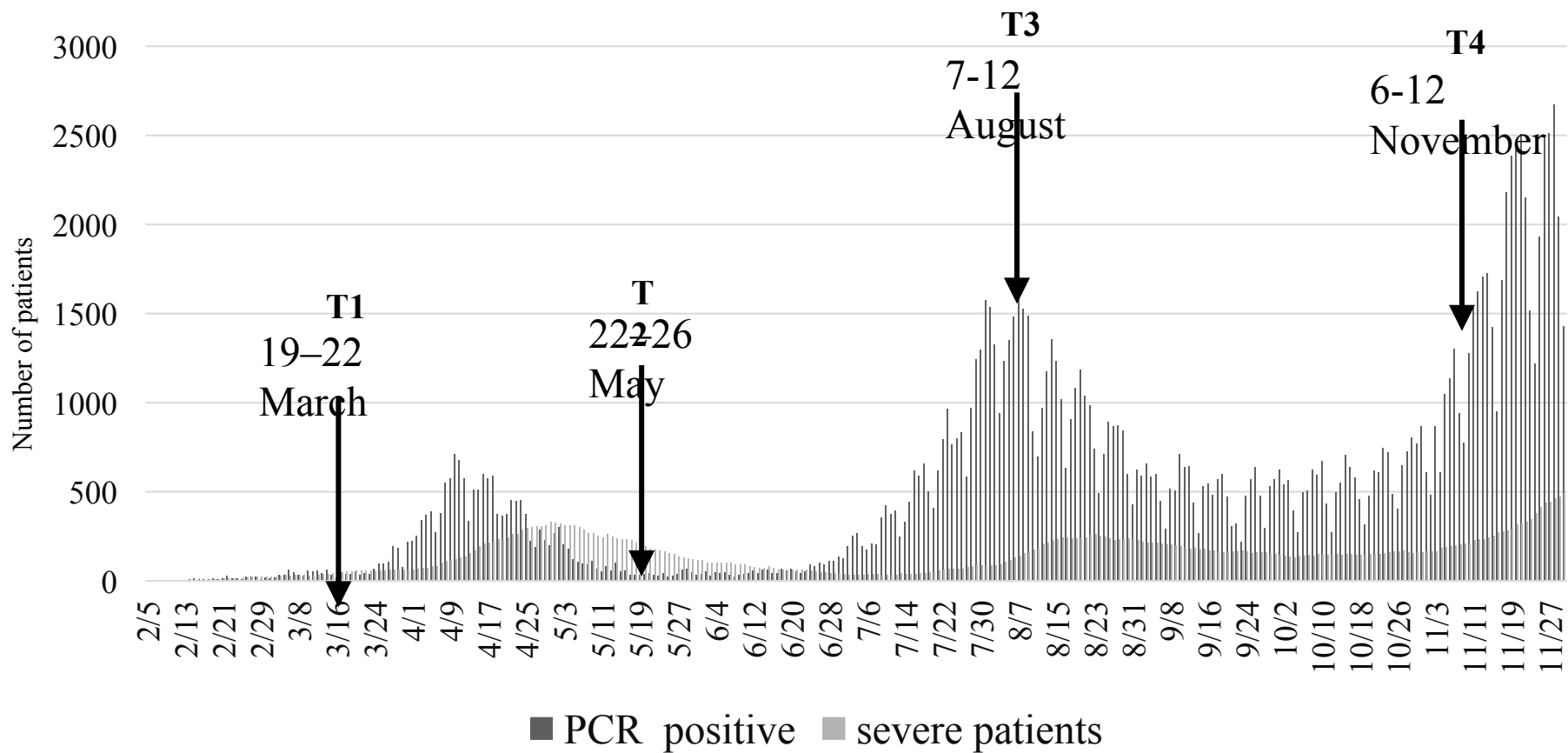

Supplementary figure2. The time point of the survey and COVID-19 situations in Japan in 2020.

Supplement: Supplementary file 3 — Supplementary Materials [file JOH2-63-e12227-s001.pdf]
