# Supplementary material for: Sustained poor mental health among healthcare workers in COVID‐19 pandemic: A longitudinal analysis of the four‐wave panel survey over 8 months in Japan
Source: J Occup Health. 2021 May 22;63(1):e12227. doi: 10.1002/1348-9585.12227 (PMC8140377; doi:10.1002/1348-9585.12227)
Supplement: Supplementary file 4 — Fig S1 [file JOH2-63-e12227-s003.pptx]

## Slide 1
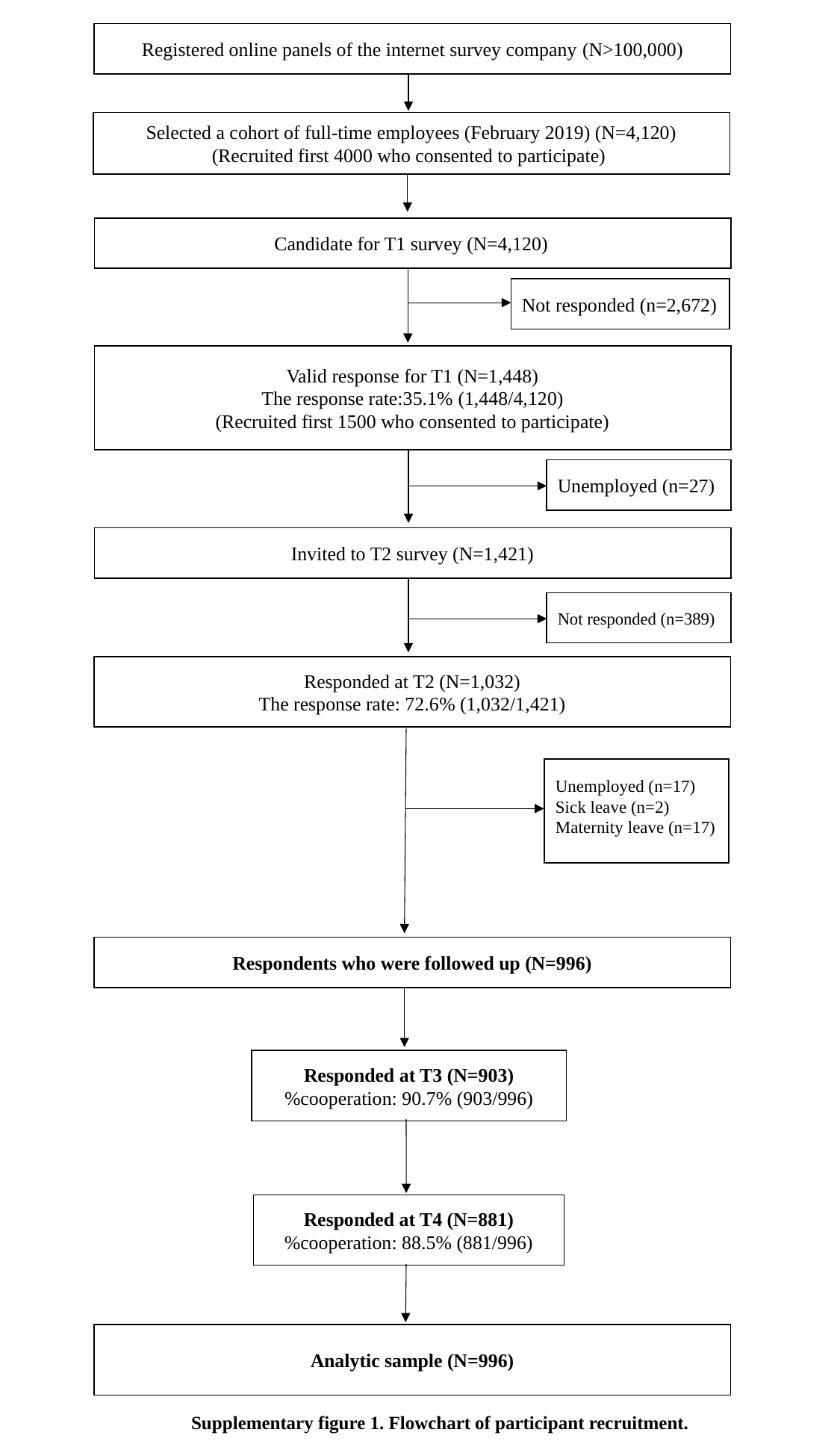

Registered online panels of the internet survey company (N>100,000)
Selected a cohort of full-time employees (February 2019) (N=4,120)
(Recruited first 4000 who consented to participate)
Candidate for T1 survey (N=4,120)
Not responded (n=2,672)
Valid response for T1 (N=1,448)
The response rate:35.1% (1,448/4,120)
(Recruited first 1500 who consented to participate)
Unemployed (n=27)
Invited to T2 survey (N=1,421)
Not responded (n=389)
Responded at T2 (N=1,032)
The response rate: 72.6% (1,032/1,421)
Unemployed (n=17)
Sick leave (n=2)
Maternity leave (n=17)
Respondents who were followed up (N=996)
Responded at T3 (N=903)
%cooperation: 90.7% (903/996)
Responded at T4 (N=881)
%cooperation: 88.5% (881/996)
Analytic sample (N=996)
Supplementary figure 1. Flowchart of participant recruitment.
