# Supplementary material for: Sustained poor mental health among healthcare workers in COVID‐19 pandemic: A longitudinal analysis of the four‐wave panel survey over 8 months in Japan
Source: J Occup Health. 2021 May 22;63(1):e12227. doi: 10.1002/1348-9585.12227 (PMC8140377; doi:10.1002/1348-9585.12227)
Supplement: Supplementary file 5 — Fig S2 [file JOH2-63-e12227-s005.pptx]

## Slide 1
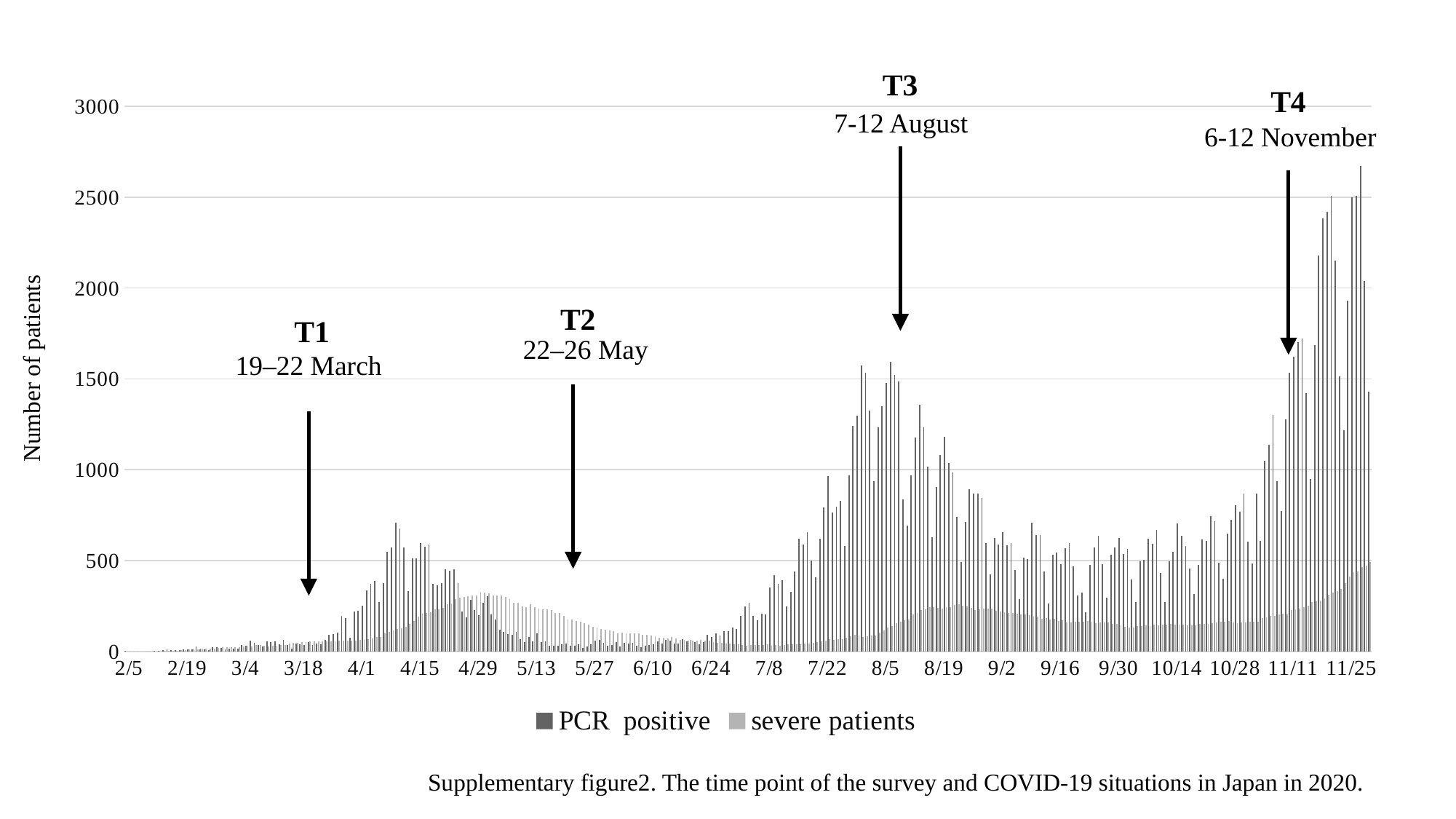

T3
T4
### Chart
| Category | PCR positive | severe patients |
|---|---|---|
| 2/5 | 2.0 | 0.0 |
| 2/6 | 0.0 | 0.0 |
| 2/7 | 0.0 | 0.0 |
| 2/8 | 0.0 | 0.0 |
| 2/9 | 0.0 | 0.0 |
| 2/10 | 0.0 | 0.0 |
| 2/11 | 0.0 | 0.0 |
| 2/12 | 1.0 | 0.0 |
| 2/13 | 4.0 | 0.0 |
| 2/14 | 7.0 | 2.0 |
| 2/15 | 12.0 | 3.0 |
| 2/16 | 6.0 | 3.0 |
| 2/17 | 7.0 | 3.0 |
| 2/18 | 7.0 | 6.0 |
| 2/19 | 10.0 | 7.0 |
| 2/20 | 9.0 | 9.0 |
| 2/21 | 11.0 | 10.0 |
| 2/22 | 27.0 | 11.0 |
| 2/23 | 12.0 | 13.0 |
| 2/24 | 12.0 | 14.0 |
| 2/25 | 8.0 | 15.0 |
| 2/26 | 22.0 | 16.0 |
| 2/27 | 24.0 | 20.0 |
| 2/28 | 20.0 | 22.0 |
| 2/29 | 9.0 | 23.0 |
| 3/1 | 15.0 | 23.0 |
| 3/2 | 14.0 | 24.0 |
| 3/3 | 16.0 | 24.0 |
| 3/4 | 33.0 | 27.0 |
| 3/5 | 31.0 | 30.0 |
| 3/6 | 59.0 | 28.0 |
| 3/7 | 47.0 | 33.0 |
| 3/8 | 33.0 | 33.0 |
| 3/9 | 26.0 | 31.0 |
| 3/10 | 54.0 | 26.0 |
| 3/11 | 52.0 | 29.0 |
| 3/12 | 55.0 | 32.0 |
| 3/13 | 40.0 | 35.0 |
| 3/14 | 62.0 | 36.0 |
| 3/15 | 33.0 | 41.0 |
| 3/16 | 15.0 | 46.0 |
| 3/17 | 44.0 | 46.0 |
| 3/18 | 39.0 | 49.0 |
| 3/19 | 36.0 | 50.0 |
| 3/20 | 53.0 | 55.0 |
| 3/21 | 34.0 | 57.0 |
| 3/22 | 42.0 | 54.0 |
| 3/23 | 38.0 | 55.0 |
| 3/24 | 65.0 | 57.0 |
| 3/25 | 93.0 | 56.0 |
| 3/26 | 96.0 | 57.0 |
| 3/27 | 104.0 | 60.0 |
| 3/28 | 194.0 | 59.0 |
| 3/29 | 185.0 | 59.0 |
| 3/30 | 74.0 | 59.0 |
| 3/31 | 218.0 | 60.0 |
| 4/1 | 224.0 | 62.0 |
| 4/2 | 253.0 | 64.0 |
| 4/3 | 337.0 | 69.0 |
| 4/4 | 370.0 | 70.0 |
| 4/5 | 386.0 | 79.0 |
| 4/6 | 270.0 | 80.0 |
| 4/7 | 377.0 | 99.0 |
| 4/8 | 550.0 | 109.0 |
| 4/9 | 572.0 | 117.0 |
| 4/10 | 708.0 | 122.0 |
| 4/11 | 676.0 | 129.0 |
| 4/12 | 571.0 | 135.0 |
| 4/13 | 333.0 | 152.0 |
| 4/14 | 511.0 | 168.0 |
| 4/15 | 511.0 | 193.0 |
| 4/16 | 596.0 | 207.0 |
| 4/17 | 575.0 | 211.0 |
| 4/18 | 590.0 | 217.0 |
| 4/19 | 372.0 | 231.0 |
| 4/20 | 365.0 | 232.0 |
| 4/21 | 374.0 | 241.0 |
| 4/22 | 450.0 | 259.0 |
| 4/23 | 445.0 | 263.0 |
| 4/24 | 451.0 | 287.0 |
| 4/25 | 374.0 | 296.0 |
| 4/26 | 221.0 | 300.0 |
| 4/27 | 189.0 | 305.0 |
| 4/28 | 284.0 | 306.0 |
| 4/29 | 227.0 | 308.0 |
| 4/30 | 199.0 | 328.0 |
| 5/1 | 266.0 | 324.0 |
| 5/2 | 302.0 | 321.0 |
| 5/3 | 204.0 | 308.0 |
| 5/4 | 177.0 | 309.0 |
| 5/5 | 120.0 | 308.0 |
| 5/6 | 106.0 | 300.0 |
| 5/7 | 96.0 | 287.0 |
| 5/8 | 89.0 | 266.0 |
| 5/9 | 108.0 | 267.0 |
| 5/10 | 68.0 | 249.0 |
| 5/11 | 50.0 | 243.0 |
| 5/12 | 80.0 | 259.0 |
| 5/13 | 54.0 | 245.0 |
| 5/14 | 100.0 | 237.0 |
| 5/15 | 52.0 | 232.0 |
| 5/16 | 57.0 | 230.0 |
| 5/17 | 30.0 | 228.0 |
| 5/18 | 30.0 | 213.0 |
| 5/19 | 31.0 | 210.0 |
| 5/20 | 38.0 | 195.0 |
| 5/21 | 43.0 | 176.0 |
| 5/22 | 31.0 | 174.0 |
| 5/23 | 29.0 | 168.0 |
| 5/24 | 40.0 | 165.0 |
| 5/25 | 20.0 | 155.0 |
| 5/26 | 27.0 | 147.0 |
| 5/27 | 37.0 | 136.0 |
| 5/28 | 61.0 | 131.0 |
| 5/29 | 64.0 | 123.0 |
| 5/30 | 46.0 | 119.0 |
| 5/31 | 32.0 | 115.0 |
| 6/1 | 36.0 | 113.0 |
| 6/2 | 51.0 | 101.0 |
| 6/3 | 26.0 | 102.0 |
| 6/4 | 45.0 | 98.0 |
| 6/5 | 41.0 | 99.0 |
| 6/6 | 45.0 | 98.0 |
| 6/7 | 32.0 | 99.0 |
| 6/8 | 22.0 | 92.0 |
| 6/9 | 30.0 | 91.0 |
| 6/10 | 36.0 | 88.0 |
| 6/11 | 40.0 | 82.0 |
| 6/12 | 57.0 | 76.0 |
| 6/13 | 43.0 | 73.0 |
| 6/14 | 62.0 | 71.0 |
| 6/15 | 60.0 | 80.0 |
| 6/16 | 42.0 | 70.0 |
| 6/17 | 43.0 | 62.0 |
| 6/18 | 68.0 | 62.0 |
| 6/19 | 54.0 | 61.0 |
| 6/20 | 65.0 | 59.0 |
| 6/21 | 49.0 | 59.0 |
| 6/22 | 40.0 | 62.0 |
| 6/23 | 53.0 | 60.0 |
| 6/24 | 89.0 | 58.0 |
| 6/25 | 79.0 | 51.0 |
| 6/26 | 99.0 | 48.0 |
| 6/27 | 88.0 | 45.0 |
| 6/28 | 111.0 | 43.0 |
| 6/29 | 110.0 | 42.0 |
| 6/30 | 132.0 | 40.0 |
| 7/1 | 125.0 | 37.0 |
| 7/2 | 194.0 | 33.0 |
| 7/3 | 249.0 | 32.0 |
| 7/4 | 268.0 | 33.0 |
| 7/5 | 195.0 | 34.0 |
| 7/6 | 172.0 | 36.0 |
| 7/7 | 208.0 | 35.0 |
| 7/8 | 203.0 | 38.0 |
| 7/9 | 352.0 | 31.0 |
| 7/10 | 420.0 | 33.0 |
| 7/11 | 373.0 | 32.0 |
| 7/12 | 391.0 | 34.0 |
| 7/13 | 248.0 | 40.0 |
| 7/14 | 327.0 | 38.0 |
| 7/15 | 440.0 | 37.0 |
| 7/16 | 619.0 | 39.0 |
| 7/17 | 588.0 | 41.0 |
| 7/18 | 655.0 | 43.0 |
| 7/19 | 501.0 | 47.0 |
| 7/20 | 407.0 | 52.0 |
| 7/21 | 618.0 | 55.0 |
| 7/22 | 792.0 | 59.0 |
| 7/23 | 966.0 | 68.0 |
| 7/24 | 766.0 | 64.0 |
| 7/25 | 798.0 | 66.0 |
| 7/26 | 830.0 | 67.0 |
| 7/27 | 581.0 | 76.0 |
| 7/28 | 968.0 | 81.0 |
| 7/29 | 1242.0 | 90.0 |
| 7/30 | 1297.0 | 87.0 |
| 7/31 | 1574.0 | 80.0 |
| 8/1 | 1535.0 | 83.0 |
| 8/2 | 1324.0 | 87.0 |
| 8/3 | 937.0 | 88.0 |
| 8/4 | 1234.0 | 104.0 |
| 8/5 | 1350.0 | 115.0 |
| 8/6 | 1479.0 | 131.0 |
| 8/7 | 1595.0 | 140.0 |
| 8/8 | 1523.0 | 156.0 |
| 8/9 | 1486.0 | 162.0 |
| 8/10 | 836.0 | 171.0 |
| 8/11 | 693.0 | 177.0 |
| 8/12 | 969.0 | 203.0 |
| 8/13 | 1176.0 | 211.0 |
| 8/14 | 1356.0 | 229.0 |
| 8/15 | 1234.0 | 232.0 |
| 8/16 | 1017.0 | 243.0 |
| 8/17 | 630.0 | 243.0 |
| 8/18 | 904.0 | 239.0 |
| 8/19 | 1080.0 | 237.0 |
| 8/20 | 1182.0 | 243.0 |
| 8/21 | 1036.0 | 243.0 |
| 8/22 | 985.0 | 254.0 |
| 8/23 | 739.0 | 259.0 |
| 8/24 | 491.0 | 252.0 |
| 8/25 | 712.0 | 246.0 |
| 8/26 | 893.0 | 238.0 |
| 8/27 | 867.0 | 227.0 |
| 8/28 | 870.0 | 230.0 |
| 8/29 | 844.0 | 234.0 |
| 8/30 | 598.0 | 236.0 |
| 8/31 | 425.0 | 234.0 |
| 9/1 | 624.0 | 225.0 |
| 9/2 | 589.0 | 218.0 |
| 9/3 | 656.0 | 214.0 |
| 9/4 | 583.0 | 210.0 |
| 9/5 | 598.0 | 211.0 |
| 9/6 | 447.0 | 209.0 |
| 9/7 | 288.0 | 202.0 |
| 9/8 | 514.0 | 202.0 |
| 9/9 | 507.0 | 198.0 |
| 9/10 | 709.0 | 191.0 |
| 9/11 | 639.0 | 190.0 |
| 9/12 | 641.0 | 180.0 |
| 9/13 | 439.0 | 185.0 |
| 9/14 | 265.0 | 174.0 |
| 9/15 | 531.0 | 178.0 |
| 9/16 | 543.0 | 167.0 |
| 9/17 | 480.0 | 170.0 |
| 9/18 | 569.0 | 161.0 |
| 9/19 | 597.0 | 159.0 |
| 9/20 | 469.0 | 163.0 |
| 9/21 | 307.0 | 164.0 |
| 9/22 | 322.0 | 165.0 |
| 9/23 | 216.0 | 166.0 |
| 9/24 | 477.0 | 163.0 |
| 9/25 | 570.0 | 156.0 |
| 9/26 | 638.0 | 160.0 |
| 9/27 | 478.0 | 159.0 |
| 9/28 | 294.0 | 161.0 |
| 9/29 | 531.0 | 151.0 |
| 9/30 | 570.0 | 151.0 |
| 10/1 | 623.0 | 145.0 |
| 10/2 | 537.0 | 137.0 |
| 10/3 | 564.0 | 133.0 |
| 10/4 | 395.0 | 131.0 |
| 10/5 | 271.0 | 140.0 |
| 10/6 | 497.0 | 141.0 |
| 10/7 | 505.0 | 143.0 |
| 10/8 | 622.0 | 141.0 |
| 10/9 | 594.0 | 147.0 |
| 10/10 | 669.0 | 145.0 |
| 10/11 | 432.0 | 146.0 |
| 10/12 | 273.0 | 148.0 |
| 10/13 | 494.0 | 150.0 |
| 10/14 | 549.0 | 146.0 |
| 10/15 | 703.0 | 148.0 |
| 10/16 | 635.0 | 148.0 |
| 10/17 | 579.0 | 145.0 |
| 10/18 | 457.0 | 144.0 |
| 10/19 | 315.0 | 143.0 |
| 10/20 | 477.0 | 150.0 |
| 10/21 | 616.0 | 150.0 |
| 10/22 | 610.0 | 151.0 |
| 10/23 | 745.0 | 155.0 |
| 10/24 | 718.0 | 159.0 |
| 10/25 | 488.0 | 162.0 |
| 10/26 | 401.0 | 165.0 |
| 10/27 | 649.0 | 166.0 |
| 10/28 | 724.0 | 161.0 |
| 10/29 | 804.0 | 156.0 |
| 10/30 | 769.0 | 161.0 |
| 10/31 | 868.0 | 160.0 |
| 11/1 | 606.0 | 163.0 |
| 11/2 | 482.0 | 163.0 |
| 11/3 | 868.0 | 165.0 |
| 11/4 | 607.0 | 183.0 |
| 11/5 | 1049.0 | 189.0 |
| 11/6 | 1137.0 | 194.0 |
| 11/7 | 1302.0 | 196.0 |
| 11/8 | 938.0 | 204.0 |
| 11/9 | 772.0 | 208.0 |
| 11/10 | 1278.0 | 204.0 |
| 11/11 | 1535.0 | 226.0 |
| 11/12 | 1623.0 | 231.0 |
| 11/13 | 1704.0 | 234.0 |
| 11/14 | 1723.0 | 243.0 |
| 11/15 | 1423.0 | 251.0 |
| 11/16 | 948.0 | 272.0 |
| 11/17 | 1686.0 | 276.0 |
| 11/18 | 2179.0 | 280.0 |
| 11/19 | 2383.0 | 291.0 |
| 11/20 | 2418.0 | 313.0 |
| 11/21 | 2508.0 | 323.0 |
| 11/22 | 2150.0 | 331.0 |
| 11/23 | 1513.0 | 345.0 |
| 11/24 | 1217.0 | 376.0 |
| 11/25 | 1930.0 | 410.0 |
| 11/26 | 2499.0 | 435.0 |
| 11/27 | 2510.0 | 440.0 |
| 11/28 | 2674.0 | 462.0 |
| 11/29 | 2041.0 | 472.0 |
| 11/30 | 1429.0 | 493.0 |7-12 August
6-12 November
T2
T1
22–26 May
19–22 March
Number of patients
Supplementary figure2. The time point of the survey and COVID-19 situations in Japan in 2020.
