# Supplementary material for: Sustained poor mental health among healthcare workers in COVID‐19 pandemic: A longitudinal analysis of the four‐wave panel survey over 8 months in Japan
Source: J Occup Health. 2021 May 22;63(1):e12227. doi: 10.1002/1348-9585.12227 (PMC8140377; doi:10.1002/1348-9585.12227)
Supplement: Supplementary file 6 — Table S1 [file JOH2-63-e12227-s004.docx]

**Supplementary Table S1. Baseline (T1) characteristics of participants (N=996).**

|  | Healthcare Workers (HCWs) ^a^  (n=111) | | Non- HCWs^a^  (n=885) | | P for difference ^b^ |
| --- | --- | --- | --- | --- | --- |
|  | N (%) | Mean (SD) | N (%) | Mean (SD) |  |
| Gender |  |  |  |  | <0.001 |
| Male | 39 (35.1) |  | 469 (53.0) |  |  |
| Female | 72 (64.9) |  | 416 (47.0) |  |  |
| Age |  | 39.6 (10.6) |  | 41.5 (10.5) | 0.078 |
| 20-29 years old | 24 (21.6) |  | 162 (18.3) |  | 0.323 |
| 30-39 years old | 36 (32.4) |  | 234 (26.4) |  |  |
| 40-49 years old | 24 (21.6) |  | 236 (26.7) |  |  |
| Over 50 years old | 27 (24.3) |  | 253 (28.6) |  |  |
| Marital status |  |  |  |  | 0.797 |
| Single | 57 (51.4) |  | 443 (50.1) |  |  |
| Married | 54 (48.6) |  | 442 (49.9) |  |  |
| Education attainment ^c^ |  |  |  |  | <0.001 |
| Junior high school | 0 |  | 7 (0.8) |  |  |
| High school | 16 (14.4) |  | 216 (24.4) |  |  |
| Vocational/College | 56 (50.5) |  | 182 (20.6) |  |  |
| University | 36 (32.4) |  | 429 (48.5) |  |  |
| Graduate university | 3 (2.7) |  | 51 (5.8) |  |  |
| Company size |  |  |  |  | 0.004 |
| >1000 employees | 22 (19.8) |  | 301 (34.0) |  |  |
| 300-999 | 28 (25.2) |  | 146 (16.5) |  |  |
| 50-299 | 30 (27.0) |  | 237 (26.8) |  |  |
| <50 | 31 (27.9) |  | 173 (19.5) |  |  |
| unknown | 0 |  | 28 (3.2) |  |  |
| Healthcare worker details |  |  |  |  |  |
| Physicians | 4 (3.6) |  |  |  |  |
| Nurses/midwives | 15 (13.5) |  |  |  |  |
| Other healthcare workers (e.g., pharmacists, clinical laboratory technicians) | 61 (55.0) |  |  |  |  |
| Health care workers but not working in clinical settings | 31 (27.9) |  |  |  |  |

SD: standard deviation.

^a^ The information about health care workers or general workers was measured on T2. Health care workers included physicians, nurses, midwives, other health care workers (e.g., pharmacists, clinical laboratory technicians), and health care workers but not working in clinical settings.

^b^ P value for difference was calculated by chi square test for variables except for age. T test was used for age.

^C^ The education attainment was measured at T2.
